# Emotional Contagion: Research on the Influencing Factors of Social Media Users' Negative Emotional Communication During the COVID-19 Pandemic

**DOI:** 10.3389/fpsyg.2022.931835

**Published:** 2022-07-15

**Authors:** Dan Lu, Dian Hong

**Affiliations:** ^1^Institute of Advanced Studies in Humanities and Social Sciences, Beijing Normal University at Zhuhai, Zhuhai, China; ^2^School of Arts and Communication, Beijing Normal University at Zhuhai, Zhuhai, China

**Keywords:** emotion contagion theory, negative emotional communication, social media, the COVID-19 epidemic, psychological mechanisms

## Abstract

During the epidemic, social media platforms were frequently used by users to express and spread negative emotions. Under emotional contagion, individual emotions gradually generalized into group emotions. At the same time, the public could not regulate their emotions and lacked access to release them rationally. This study explores the factors influencing the negative emotions' communication among social media users during the COVID-19 epidemic from the perspective of emotion contagion theory to discover the psychological mechanisms among the public. The questionnaire was tested for reliability and validity and then distributed online on Chinese social media platforms, and the data collected were statistically analyzed. The findings show that there are significant differences in negative emotional communication in social media among different age groups; the seven dimensions of deindividuation, risk perception, group identity, group efficacy, event stimulation, event publicness, and emotion contagion all have significant positive effects on users' negative emotional communication. This study aims to raise public awareness of negative emotions and promote the reconstruction and recovery of public mental health in the epidemic era.

## Introduction

Since the outbreak of the COVID-19 in 2019, the epidemic has suddenly threatened life safety and physical health of the public. With the changing and repeated situation of the development of the epidemic, the mental health of the public has become more complicated. The Blue Book on Social Mindset: 2019 China Social Mindset Research Report, released by the Institute of Social Sciences in December 2019, mentioned that the public significantly reduced the control point and tolerance level of social emotions under frequent social events during the new coronavirus epidemic (Wang and Chen, 2019). The social events as a trigger, together with the psychological stress and anxiety under the epidemic, made social emotions inclined to negative emotions such as anger, resentment, and pain.

During the COVID-19 pandemic lockdown, Internet platforms, such as online communities accessible to users, have become a convenient and open place for public emotions to be expressed. Any hot event may quickly create a buzz on social media platforms such as Weibo, WeChat, and TikTok, generating millions of topics attention and discussion. Importantly, in an information-explosive media environment, public sentiment has a much more significant impact on public opinion than the event. Emotion contagion theory suggests that emotions can spread rapidly in online communities and can be accepted and perceived by others with the same feelings, gathering a large community of emotions in a short period through the characteristics of emotion (Brian, 2019), which may result in mass anger and uncontrolled public opinion in cyberspace.

The hot events under the COVID-19 epidemic, such as the topic of most significant concern to the public, have constructed a public opinion field loaded with the interests and emotional needs of various groups. In contrast, public generally exists in a negative mood, overloading the entire public opinion environment with negative emotions that backfire on the public. This is because emotions are not only private but also a social resource. In the society, the COVID-19 epidemic remains a vast and severe challenge, and there is not only the spread of the virus to be faced but also an information epidemic, harmful emotion infection, and other potential problems that need to be addressed. From the perspective of individual mental health, emotions are not just a state of mind but a resource that can influence society and others. When the public's living space is filled with negative emotions and the effects of emotional contagion go unnoticed, it can severely impact an individual's mental health, exacerbating negative emotions such as anxiety and fear. Society will remain under the gloom of the epidemic for a long time.

Based on the context of public psychological reconstruction and recovery in the post-epidemic era, this study introduces emotional contagion variables to investigate the factors influencing the negative emotions' communication among Internet users during the epidemic, to provide some reference for the causes of negative public emotions and ways to channel them under major public health events.

## Literature Review

### Emotional Contagion Theory

Emotional contagion originated in psychology and is defined as “a direct primitive response produced by sympathetic nerves in the brain.” While early research considered emotion contagion as unconscious imitative behavior, later research has argued that emotion infection is a conscious process and is subject to conscious regulation. In the course of the debate, mechanisms for emotional infection have also been constructed, such as imitation-feedback mechanisms, associative-learning mechanisms, and social comparison mechanisms (Zhang and Lu, 2013). The imitation-feedback mechanism suggests that emotional infection consists of three stages: imitation, feedback, and infection. The association-learning mechanism refers to an observer displaying emotions similar to those of others when they are in the same situation as others and are induced by the emotions of others. Under the effect of emotional contagion, it is possible to predict how the behavior of organizations will affect individuals and how they will react (Gustave Le, 1895). After much social research by many scholars, emotional contagion has been widely defined as an emotional experience: “People capture and feel emotions from messages transmitted by others and subconsciously imitate and feel them, thus exhibiting similar emotions.” The characteristics of emotional contagion are (i) interactivity: emotional contagion must occur in the process of interpersonal communication; (ii) directionality: emotional contagion is directional, and there must be a transmitter and a receiver in the process; (iii) similarity: similarity is an essential characteristic of emotional contagion, the receiver of the emotion will have similar emotional experiences and expressions as the transmitter in the process of emotional infection, and eventually the emotions of both converge (Herrando and Constantinides, 2021).

Research on the influencing factors and mechanisms of emotional infection: emotional infection is influenced by the infected person's perceived ability, emotional state, attentional tendencies, and values. The mechanisms by which emotional infection occurs are as follows: emotional information is delivered → , perceived by the receiver → , unconscious imitation by the receiver → , physiological feedback → emotional experience (Zhang, 2014). The mechanism of emotion contagion during emergencies shows that increasing the size of negative emotion groups will increase the time of group emotion contagion and improving the ability of individuals to recover themselves can shorten the time of group emotion contagion spreading and make group emotion enter the decline period earlier, which is conducive to the control and regulation of group emotion (Chen et al., 2018).

Negative emotions in social media can lead to emotional contagion and that infected individuals can quickly turn into negative information spreaders. Improving negative emotions in social media is essential to mitigate disinformation, guide public opinion, and comply with policies, such as vaccination during pandemics (Yin et al., 2022). Negative emotions were mainly generated by fear and worry about the epidemic and doubts about the work of the government. Second, there is a strong correlation between people's emotions and changes in the epidemic. In addition, the study found that women had more positive emotional displays than men (Pan et al., 2021). Therefore, the communication of negative emotions among groups with different demographic characteristics deserves further research.

Research on the contagious effects of emotions suggests that non-verbal symbols—social media emojis can convey emotions, interact with other groups, and create environments and atmospheres. Users can share their emotional experiences and interact and share emotions through non-verbal symbols on the Internet. Emotional contagion can have the effect of creating emotional resonance among group members. Individuals can build emotional ties and a sense of group solidarity in emotional contagion and eventually construct emotional memories (Gu, 2018). When negative emotions are highly contagious, they tend to interfere with the ability to regulate mental health and induce inappropriate behavior to occur. Negative emotions have a positive and significant impact on irrational behavior, and emotional contagion plays a partially mediating role in the generation of negative emotions.

Therefore, when emotional stress increases, the government should pay special attention to the mental health environment (Petitta et al., 2021). During the epidemic outbreak, the proportion of negative sentiment on Twitter gradually increased and was amplified following actual events. Internet found increasingly hostile sentiment scores by observing active social media users, and the negative feeling persisted. Therefore, monitoring social media like Twitter can stop sentiment contagion and is a new way to prevent information epidemics (Crocamo et al., 2021).

### Negative Emotional Communication

In the early 20th century, positive and negative emotions were separate concepts and phenomena in empirical studies in psychology. Psychologist Watson D et al. identified 20 words to measure emotions, 10 of which were used to measure negative emotions. The Emotion Scale has been widely used to assess mental health in psychotherapy after passing validity and applicability tests. Eventually, psychology referred to sadness, upset, guilt, fear, hostility, anxiety, shame, tension, anger, and frustration collectively as negative emotions (PANAS; Watson et al., 1988). Negative emotional communication is the interactive process in which the information carried in the process of emotion communication is negative emotion information, and the communicator experiences and shares the negative emotion information with other individuals. Negative emotional information originates when communicators perceive, evaluate, and react to external stimuli based on their own emotional experiences and transform feelings into externalized messages when negative emotional experiences exceed tolerance thresholds (Liu et al., 2018).

Research on the propagation patterns and dynamics of negative emotions shows that emotional communication is influenced by triggering events, group identity, closeness and trust, social support, interpersonal interaction, and a sense of belonging. When group members are more closely related, the higher possibility of group emotional communication, the faster they spread, and the more widespread. In group emotion communication, the number of communicators and the intensity of emotions within the group tend to increase and then decrease over time. It has been found that even when group emotions cease to spread, the intensity of group emotions does not decrease simultaneously. Therefore, if a group event or group sentiment is not handled effectively or if vigilance is relaxed, there is a high risk that group sentiment will return (Wang et al., 2014). A study of the presentation of emotions in social media has revealed an “emotion preference” in spreading emotions. Angry emotions are more numerous and have a higher weight than other emotions. When anger was the dominant emotion, the concentration of online emotions shifted toward anger. There is a stronger positive correlation between the intensity of emotions in news messages and anger than positive emotions; there is also a stronger positive correlation between the popularity of news and anger (Xu, 2017).

In public issues, the stimulation and emotional contagion of unjust and immoral events can trigger negative emotions such as anxiety, anger, and sadness. When anger and despair are ignited, it can lead to a rise in altruistic tendencies. For the group, external pressures will encourage the group to become more united and supportive (Chen et al., 2021). Anxiety about social safety, the spread of the virus, perceived risk of infection, and media exposure had a significant effect on the communication of negative emotions and influenced the choice of preventive behaviors. In a study of Korean adults, women, the elderly, and those with poorer self-rated health perceived more significant risk, experienced more negative emotions, and thus adopted more preventive behaviors (Kim et al., 2020). Increased use of digital media activities, online socializing, and teleconferencing during an epidemic can lead to stronger feelings of deindividuation. Higher deindividualization experiences lead to increased negative emotional interactions. Overly sedentary, digital lifestyle habits may induce an individual's sense of disconnection from the body, self, and world (Ciaunica et al., 2022). Consequently, this study suggests that the communication of negative emotions may be influenced by the variability of individuals' self-presentation on social media and multiple elements of group relationships, social events, and emotional characteristics.

### Overview of Topical Events

The primary source for selecting hot events during the COVID-19 epidemic is the Internet social hotspot aggregation platform “Zhiwei Shijian,” a team led by a postdoctor from the Chinese Academy of Sciences, which is one of the top data mining platforms in China, providing information intelligence, event analysis, and tracking digital information for enterprises and governments. A total of 400 hot events from 2019 to 2022 were selected from the annual and monthly Influence List of “Zhiwei Shijian,” and eight public events that network users highly discussed during the epidemic were listed concerning the research theme (Table 1). The Event Influence Index (EII) is an authoritative indicator of the impact of a single event on the Internet based on self-published and online media data from across the web. Table 1 is ranked in descending order of influence.

**Table 1:** Hot events during the outbreak.

**Time**	**Events**	**EII**
2019.12.30	COVID-19 outbreak in Wuhan and other places and human-to-human transmission	103.6
2022.03.01	Confirmed local cases resurface in Shanghai, increasing by tens of thousands daily.	96.3
2020.02.06	Epidemic whistleblower Dr. Li Wenliang dies of COVID-19.	84.8
2020.01.23	Lockdown on Wuhan.	84.5
2020.01.30	Questions arise over the use of supplies by the Hubei Red Cross.	84.3
2022.01.04	A pregnant woman in Xi'an had a miscarriage after waiting 2 h in front of the hospital.	80.6
2022.03.30	Residents need to grab food online during the Shanghai closure, and the elderly are short of supplies.	73.6
2022.02.15	Gansu nurses supporting Hubei asked for hair shaving causing controversy.	64.1

The events listed in the table above are a part of topical events in China during the COVID-19 epidemic. The cases have similarities in that (i) they conflict with the values of netizens, such as the miscarriage of a pregnant woman, the difficulty of buying groceries for the elderly, and the death of Dr. Li Wenliang; and (ii) they have a wide range of impacts and long duration, such as the lockdown of Wuhan and Shanghai for city management. The cases all sparked a great deal of discussion on social media platforms, with their specific content presenting a rise from criticism of the government to mutual attacks between people from different regions. Negative sentiments were then ignited, emotional messages even drowned out objective issues that urgently needed to be addressed, and society was plunged into a depressed and frustrated state. This study provides a more explicit definition of the event variables in subsequent studies by outlining some of the hot events and their nature.

## Research Design

### Research Model

This study takes users of social media platforms as the research target and investigates the factors influencing users' negative emotional communication behavior in using social media platforms. Based on the research objectives and previous research frameworks, four influencing factors were selected: individual, group, event, and emotion.

**Figure 1:**
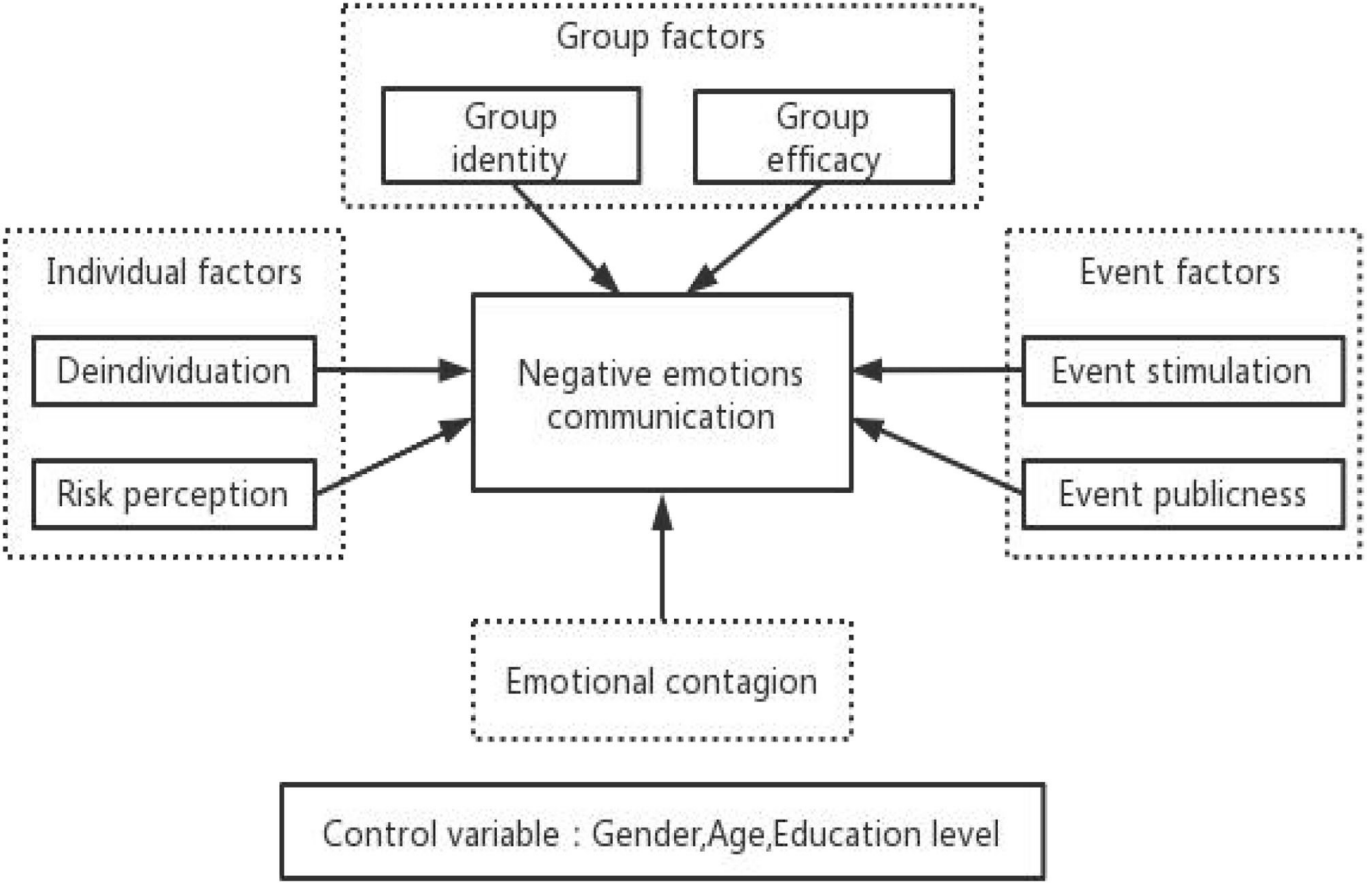
Hypothesis model.

The individual factors include two dimensions: deindividuation and risk perception. This study selected two dimensions for the group factors: group identity and group efficacy. Two measurement dimensions were chosen for the event factor: event stimulation and event publicness. Among the demographic variables, gender, age, and education level were introduced as control variables in this study. Ultimately, this study constructed the following conceptual model (Figure 1).

### Definition and Hypotheses

#### Individual Factors

Deindividuation refers to individuals being controlled by group ideology and goals, weakening their self-perception and self-orientation, resulting in a severe decline of self-control and an inability to control their behaviors (Serena et al., 2019). The main reasons why deindividuation arises are (i) anonymity: when the more an individual is hidden from the group, the lower the individual's sense of responsibility and (ii) decreased self-control: when individuals lose moral judgment and control over their own words and actions, they are prone to intense emotions and speech, which may have serious consequences (Paul et al., 2016).

Risk perception refers to an individual's perception and judgment of the attributes and severity of a particular situation and is used in psychology to measure the degree of psychological panic in individuals (Dönges et al., 2022). In the research of risk perception models, Slovic mentioned that: (i) risk perception is quantifiable and predictable; (ii) risk perception is dynamic in the time dimension; and (iii) risk perception is individually variable, with each individual assessing the same situation with different risk criteria (Paul and Ellen, 2006).

#### Group Factors

Group identity is a special relationship between group members based on agreed perceptions, attitudes, and interests. Group identity allows individuals to project feelings and values about the group they belong to and develop a sense of belonging. The main characteristics of group identity are (i) the maintenance of solidarity between group members; (ii) the attitude and behavior of group members in support of the organization; and (iii) the sharing of feelings with other members (Miyazono and Inarimori, 2021).

Group efficacy is the shared beliefs of group members about the group's ability to perform a behavior together and the achievements it produce. The establishment of group efficacy affects the choice of group behavior, the establishment of group goals, and the level of effort put into group behavior (Smith Elaine et al., 2021). When vulnerable groups encounter frustration and injustice, those with high group efficacy are more likely to be motivated to reform and feel hope. Group efficacy has implications for society's understanding of the emotional and behavioral motivations of the public in contexts of oppression (Siwar et al., 2020).

#### Event Factors

Event stimulation means that the event will involve some sensitive topics, and its stimulating properties will play a catalytic role in the occurrence and evolution of the issue. The following four conditions are met: (i) the event is public that the audience can directly perceive; (ii) the event is in line with the focus of social opinion; (iii) the event conflicts with the general values of the audience; and (iv) the event is related to the public interest (Yuan, 2015). When the occurrence of an exciting event triggers the focus of attention of social media users, the Internet era is a time when everyone is a “radio station without a license,” which dramatically increases the possibility of users spreading emotional messages (Zhang and Chen, 2012).

Event publicness refers to the extent to which an event is harmful to the public interest and generally involves topics such as the promotion of public morality, the protection of civil rights, the maintenance of public order, and the supervision of public authority. The event publicness is higher, the greater the number of people involved, the more widespread the impact, and the greater the public interest at stake. When measuring the publicness of an event, it is limited to not only the current social impact but also the potential for harm to the social system, which cannot be ignored (Yuan and Xu, 2015).

#### Emotional Contagion

When the emotions carried in a message influence and change the feelings of others, which in turn influence their behavior. By capturing data on users' discussions on Twitter during the outbreak for sentiment analysis, this study found that as users' arguments about the episode became more intense, sentiment scores became increasingly negative, and the authors proposed that emotional contagion exists in social media. Therefore, users' emotional tendencies can predict positive or negative emotional communication behavior (Crocamo et al., 2021).

#### Demographic Variables

Three of the demographic variables, gender, age, and degree of education, were included in this study to examine whether there were differences in the behavior of social media users in negative emotions' communication. The relevant study highlighted significant differences in the negative emotions' behavior across different demographic characteristics (e.g., gender, age, and degree of education). For example, in the study of epidemic prevention such as restricting outdoor activities and the development of negative emotions and irrational behavior among students, differences were confirmed across age and gender (Rezapour et al., 2022).

This study introduces the above seven dimensions and proposes the following hypotheses based on available research results.

**Hypothesis 1(H1):** There is a significant positive effect of deindividuation on users' negative emotional communication.
**Hypothesis 2(H2):** There is a significant positive effect of risk perception on users' negative emotional communication.
**Hypothesis 3(H3):** There is a significant positive effect of group identity on users' negative emotional communication.
**Hypothesis 4(H4):** There is a significant positive effect of group efficacy on users' negative emotional communication.
**Hypothesis 5(H5):** There is a significant positive effect of event stimulation on users' negative affective communication.
**Hypothesis 6(H6):** There is a significant positive effect of event publicness on users' negative emotional communication.
**Hypothesis 7(H7):** There is a significant positive effect of emotion infection on users' negative emotional communication.
**Hypothesis 8(H8):** There is no significant difference between users' negative emotional communication on social media by gender.
**Hypothesis 9(H9):** There is no significant difference between users' negative emotional communication on social media by age.
**Hypothesis 10(H10):** There is no significant difference between users with different levels of education in their social media communication.

### Measuring Instruments

When individuals are in a state of deindividuation, the greater the likelihood that they will participate in public social events. There is a difference between an individual in a group setting and an individual in a solitary state. Individuals in a group environment become less self-controlled and gradually lose their sense of self, which may be a loss of control of their behavior (Zhang and Wang, 2017). Internet users selectively receive information and intermittently vent their emotions, showing a de-individualized state of personality carnival, which is very likely to drive group emotions to extremes and cause radical group behavior, such as spreading rumors and verbal bullying. In this study, the primary reference in the design of the deindividuation scale is the Web-based DeIndividuation Scale, which consists of four dimensions of question setting: reduced self-awareness, emotional catharsis, low self-evaluation ability, and reduced level of self-control (Zheng, 2013). The questionnaires were on a 5-point Likert scale from 1 (strongly disagree) to 5 (strongly agree). The deindividuation dimension consists of four questions (e.g., “In an anonymous online environment, I would be free to express myself, whereas, in real life, I would be apprehensive.” and “I am more likely to make emotional statements to vent my emotions in an anonymous online environment than in a real-life environment”).

Emotional factors cause 63% of people to experience fluctuations in their attitudes toward risk. Anger and fear are the most pronounced types of emotion. Risk perception directly influences people's behavioral decisions and risk judgments (Meng et al., 2010). The Risk Perception Scale was designed regarding the Public Perception of Risk in Public Health Emergencies Scale, which consists of three dimensions of questions: severity of health effects, social risk, and controllability (Dai et al., 2020). The questionnaires were on a 5-point Likert scale from 1 (strongly disagree) to 5 (strongly agree). Risk perception consists of three questions (e.g., “This virus is easily infected, and I am likely to be infected.” and “I feel that the epidemic and spread of this flu is difficult to control.”)

With group identity, the emotional reactions of some people experiencing a particular event can affect other group members. When external circumstances jointly threaten the group, it increases the risk-taking nature of group behavior and causes group members to adopt more aggressive behavioral patterns (Yi and Zhang, 2015). When members have a high level of group efficacy, their willingness to participate will subsequently increase as long as members perceive group behavior as effective and beneficial. Driven by a sense of group efficacy, public opinion messages tend to spread the event's negative impact in question and amplify the spread of that type of event (Zheng, 2017). This study refers to the classic scales of group identity and group efficacy and considers the actual situation of users' negative emotional communication on social media during the epidemic to compile a scale (Xue et al., 2013). The questionnaires were on a 5-point Likert scale from 1 (strongly disagree) to 5 (strongly agree). The group factors consisted of six questions (e.g., “I can feel a sense of belonging when most users have the same attitude as me toward the epidemic.” and “I think that the large number of users expressing negative emotions about the epidemic can help defend the rights of the weak in the hot events of the epidemic”).

The occurrence of an irritating event is the triggering factor that draws otherwise dispersed individuals together to engage in a lively discussion about the current annoying event, triggering negative emotion transmission behavior (Du and Wei, 2010). The public nature plays a crucial role in whether or not it becomes the focus of online opinion. The more public an event is, the more significant the social impact it will have. The degree of publicness of an event can positively predict the occurrence of negative emotional communication (Yuan and Xu, 2015). The event stimulation and event publicity test scales are derived from well-established scales in Chinese Internet emotion-related research, both systematically tested for reliability (Lu, 2018). The questionnaires were on a 5-point Likert scale from 1 (strongly disagree) to 5 (strongly agree). The event factor consists of seven questions (e.g., “I am more willing to follow the progress of and participate in hot events in epidemics when they involve certain sensitive topics” and “I am more willing to follow the progress of and participate in hot events on the Internet when they involve a large number of people”).

In social media platforms, emotions occupy a crucial position. The mutual contagion and incitement of feelings can easily lead to irrational group emotions and act as a catalyst for emotion communication behavior (Blake et al., 2020). The Emotional Contagion Scale was developed by referring to scales related to emotional infection in social media (Song et al., 2017). The questionnaires were on a 5-point Likert scale from 1 (strongly disagree) to 5 (strongly agree). The Emotional Contagion dimension consists of three questions that follow the pattern of emotional infection, i.e., feeling, identification, and infection (e.g., “I identified with other people's emotions and felt the same way during the discussion about the epidemic,” and “I get infected by other people's emotions (anger, resentment, sympathy, etc.) during discussions about the epidemic”).

In this study, the scale design of negative emotional communication behavior mainly refers to the research literature and questions related to the evolution mechanism of online public opinion and emotional expression and the actual situation of users' negative emotional communication behavior in social media during the epidemic (Song and Chang, 2021). The questionnaires were on a 5-point Likert scale from 1 (strongly disagree) to 5 (strongly agree). The dimensions of the questions include social media use, interactive behavior in social media, and social media self-presentation (e.g., “I will express my negative emotions in social media,” “I will interact with others on social media with negative emotions (including reposting, commenting, liking, etc.),” and “Other users of social media can realize my emotional state through my behavior”).

### Participants and Procedures

Before the study was conducted, 139 samples were collected for questionnaire pre-testing to ensure the reliability and validity of the follow-up study. During the formal research phase, questionnaires were distributed online through online platforms. As this study involved users' social media usage, the questionnaire was distributed through multiple social media platforms such as Weibo, WeChat, and QQ through a snowballing process, thus expanding the scope of the sample.

About 443 questionnaires were collected, and the second question asked, “Have you ever expressed your attitude or emotion on social media (including reposting, commenting, liking, etc.).” About 51 respondents chose “never” and were therefore not included in the analysis sample for this study. About 392 questionnaires were valid.

The gender ratio of participants was roughly equal (men = 48.724%; women = 51.276%). The largest proportion of respondents was aged 18–25, at 52.040%, and the second-largest age group was aged 25–40, which is also the main force on social media platforms. Regarding the distribution of respondents' education level, the largest proportion of respondents were bachelors (51.530%) and masters and above (30.103%). The details are listed in Table 2.

**Table 2:** Demographic information results.

**Demographic variables**	**Item**	**Number**	**Percentage**
Gender	male	191	48.724
	female	201	51.276
Age	Under 18 years old	26	6.633
	18–25 years old	204	52.040
	26–30 years old	66	16.837
	31–40 years old	68	17.347
	Over 41 years old	28	7.143
Level of education	High school education or below	72	18.367
	Bachelor's degree and specialist qualifications	202	51.530
	Master's degree and above	118	30.103

## Data Analysis

### Reliability and Validity Analysis

In this study, SPSS 23.0 was used to analyze the data. Cronbach's alpha was chosen as the main criterion for the reliability analysis, and the exploratory factor analysis was used to conduct the KMO test and Bartlett's sphericity test for the validity analysis. The results are listed in Table 3. The overall validity KMO value of the scale was 0.974, the KMO value of each variable was >0.7 (sig = 0.000), the factor loadings were all above 0.7, and the extracted sum of squares was >60%, which passed the validity test.

**Table 3:** Reliability and validity analysis for each variable.

**Variable**	**Item number**	**Cronbach's alpha**	**KMO**
Negative emotions communication	3	0.880	0.734
Deindividuation	4	0.916	0.843
Risk perception	3	0.862	0.731
Group identity	3	0.884	0.737
Group efficacy	3	0.904	0.756
Event stimulation	3	0.837	0.722
Event publicness	4	0.894	0.825
Emotional contagion	3	0.868	0.735

### Descriptive Statistical Analysis

This study conducted descriptive statistical analysis for each variable. A five-point Likert scale was used in this study, ranging from 1 strongly disagree to 5 strongly agree and then calculate the mean; if the mean is >3, it indicates that the majority of people have a positive attitude toward this question, and a mean <3 showed a negative attitude. The standard deviation determines the difference between the respondents on a particular question. A more significant standard deviation indicates a tremendous difference in the respondents' answers to this question, and a smaller standard deviation indicates a minor difference. Descriptive statistics for each variable are listed in Table 4.

**Table 4:** Descriptive statistical analysis of each variable.

**Variable**	**Mean value**	**Standard deviation**	**Median**
Deindividuation	3.826	0.965	4.250
Risk perception	3.856	0.969	4.000
Group identity	3.866	0.946	4.000
Group efficacy	3.935	0.948	4.333
Event stimulation	3.938	0.884	4.000
Event publicness	3.937	0.906	4.250
Emotional contagion	3.885	0.916	4.000
Negative emotions communication	3.758	0.960	4.000

The mean of all question items is above 3.5, indicating that the respondents have a more agreeable and positive attitude toward each question item. The standard deviations are all below 1, reflecting little individual variability. The mean values of the variables group efficacy, event irritation, and event publicness are high, all above 3.9, illustrating that these three elements are perceived more strongly during users' communication of negative emotions. In particular, the median value of group efficacy also ranked first, at over 4.3, demonstrating that the public is in a state of high efficacy in negative emotional communication.

In terms of the specific questions of the variables, the third question under the group efficacy variable “I think that the large number of users expressing negative emotions about the epidemic can make the authorities move in a more people-friendly direction.” The fourth question under event publicness is, “I am more willing to follow the progress of an online hot topic and participate in it when it involves doing justice for public morality.” The mean value for both of these questions was above 4, suggesting that users may believe that spreading negative sentiment on social media platforms can promote policy-making, justice, and other positive directions.

### Descriptive Analysis of Users' Social Media Use

This study set two questions to investigate users' social media usage during the COVID-19 epidemic: (i) access to information and (ii) time spent daily on social media platforms.

**Figure 2:**
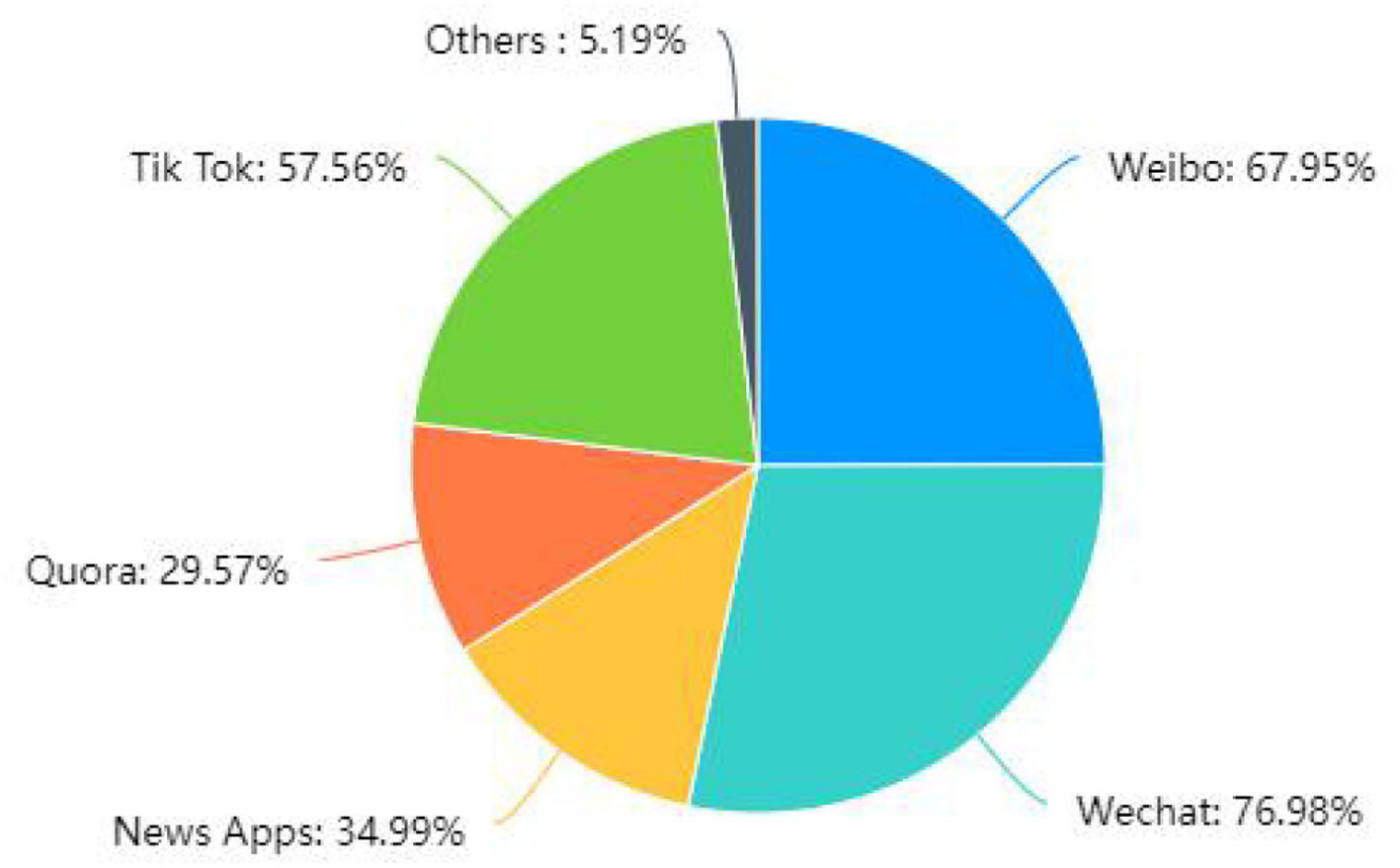
Access to information for users during the COVID-19 outbreak.

The survey results on the access to information are shown in Figure 2. The social media platform more frequently used by users during the epidemic had the highest percentage of WeChat, accounting for 76.98%, followed by Weibo, accounting for 67.95%. This study set up a fill-in-the-blank in the questionnaire, allowing users to add their own commonly used platforms, other in the figure, accounting for (5.19%), with users filling in Douban, RED, and video websites.

**Figure 3:**
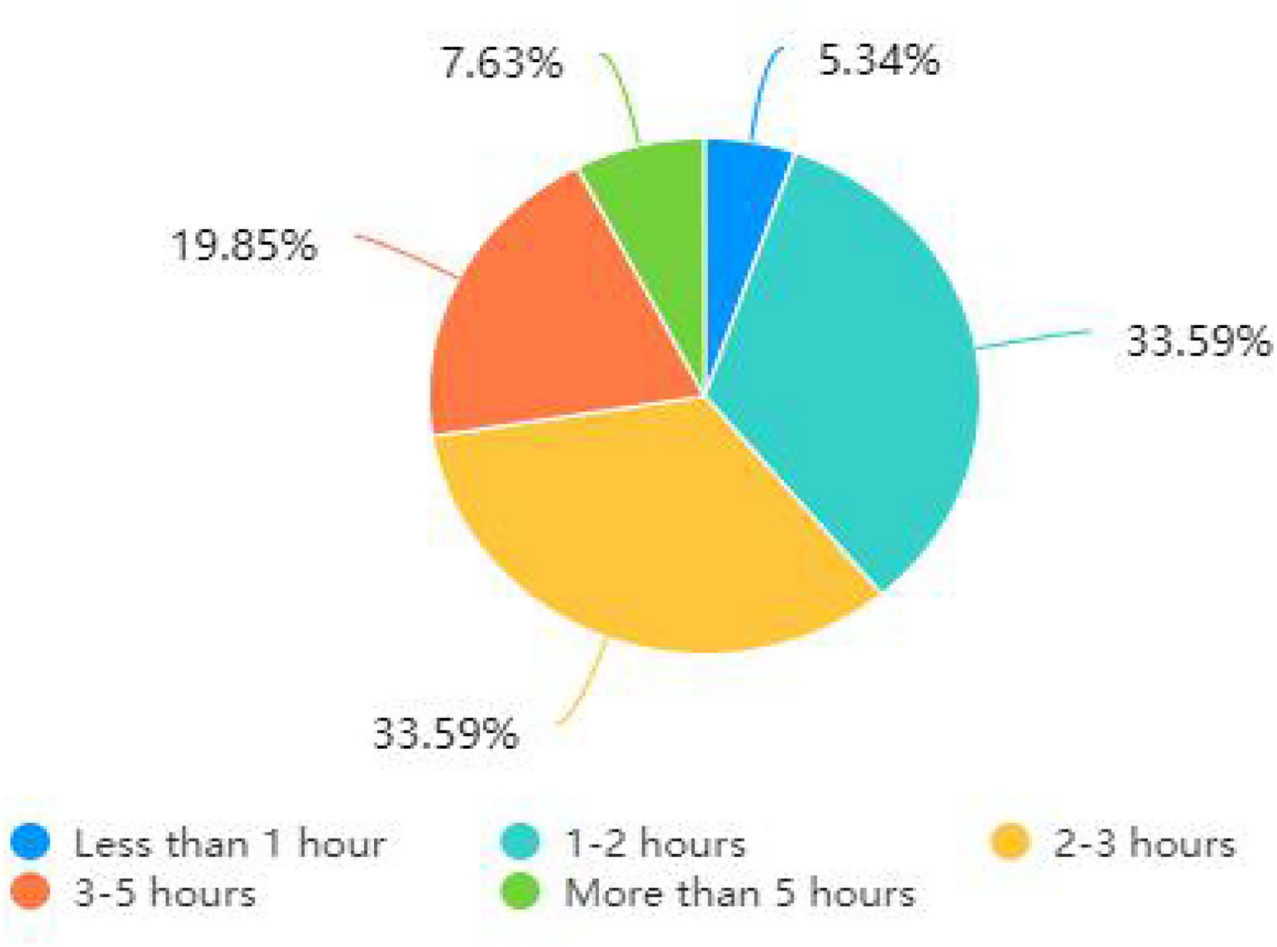
Time spent daily on social media platforms during the COVID-19 outbreak.

As shown in Figure 3, users' daily use of social media platforms was concentrated in 1–2 h and 2–3 h, with the same percentage of 33.59%, followed by 3–5 h, with 19.85%. Fewer users (5.34%) used the platform for <1 h a day. This graph shows that most users spend part of their day browsing social media platforms for information, with fewer users blocking social media information altogether.

### Variance Analysis

According to the research hypothesis, an independent sample *t-*test and one-way ANOVA test were mainly used to investigate whether there were significant differences in negative emotional communication on social media platforms among users of different genders, ages, and education levels.

The test results showed the following: (i) In the gender difference study, *t* = 0.661, *p* = 0.509 > 0.05, which did not reach a significant level, so there was no significant difference in negative emotional communication between genders. (ii) In the study of the difference in educational attainment, *F*=1.057, *p* = 0.349 > 0.05, there is no significant difference in the line of negative emotional communication by educational attainment.

In the age group difference study, *F* = 2.247, *p* = 0.038 < 0.05, indicating a significant difference between age groups for negative emotional communication. Hypothesis 9 does not hold (Table 5).

**Table 5:** ANOVA results for age on negative emotional communication.

	**Quadratic sum**	***Df***	**Mean square**	***F***	***P***
Between-group variation	12.200	6	2.033	2.247	0.038
Within-group variation	348.444	385	0.905		
Sum	360.644	391			

The research obtained further insight into the specific distribution of differences by comparing groups through Scheffe's method, which showed that the group with stronger negative emotional communication was concentrated in the 18–25 age group, followed by the 26–30 age group. Negative emotional communication was weaker among users aged 41 and above (Table 6).

**Table 6:** Mean difference results.

**Age group**	**Harmonic mean**
Under 18 years old (*n* = 26)	3.7949
18–25 years old (*n* = 204)	3.9559
26–30 years old (*n* = 66)	3.8737
31–40 years old (*n* = 68)	3.8333
Over 41 years old (*n* = 28)	3.5374

### Correlation and Regression Analysis

This study explored the correlation between the independent and dependent variables through Pearson's correlation coefficient. The results of the correlation analysis are summarized in Table 7. Specific research shows that the correlation coefficient values between negative emotional communication and deindividuation, risk perception, group identity, group efficacy, event stimulation, event publicness, and emotional contagion all fall within the 0.7–0.8 range and show a 0.01 level of significance, thus indicating that each of the respective variables has a significant positive correlation with negative emotional communication.

**Table 7:** Correlation analysis results.

	**Negative emotions communication**
Deindividuation	0.812^**^
Risk perception	0.734^**^
Group identity	0.758^**^
Group efficacy	0.796^**^
Event stimulation	0.759^**^
Event publicness	0.753^**^
Emotional contagion	0.777^**^

^*^p < 0.05;

** *p < 0.01*.

While correlation analysis describes whether there is a relationship between the analyzed terms, regression analysis explores the influence of the variables on each other. This study will further use regression analysis to understand the degree of influence between the variables and obtain a clear functional relationship.

The summary results of the model are shown in Table 8, with an adjusted *R*^2^ = 0.740, implying that emotional contagion, event publicness, event stimulation, group efficacy, group identity, risk perception, deindividuation explained 74% of the negative emotional communication, with an excellent explanatory effect. The D-W value test was 1.811, indicating that the residuals were independent and the model did not suffer from serial correlation.

**Table 8:** Model summary.

**R**	**R^**2**^**	**Adjusted R^**2**^**	**Standard error**	**DW**
0.863	0.745	0.740	0.485	1.840

The results of the ANOVA are shown in Table 9, *F* = 160.012, *p* = 0.000 < 0.05, the model passed the test that at least one of the independent variables had a significant relationship of influence on the dependent variable.

**Table 9:** ANOVA.

	**Quadratic sum**	***df***	**Mean square**	***F***	***P***
Regression	268.569	7	38.367	160.012	0.000
Residue	92.074	384	0.240		
Sum	360.644	391			

The results of the validation of the regression coefficients for each variable are as follows:

- the regression coefficient for deindividuation was 0.809 (*t* = 27.521, *p* = 0.000 < 0.01), implying that deindividuation would have a significant positive relationship with negative emotions' communication. Hypothesis 1 does not hold.
- the regression coefficient for risk perception was 0.727 (*t* = 21.356, *p* = 0.000 < 0.01), implying that risk perception would have a significant positive relationship with negative emotions' communication. Hypothesis 2 does not hold.
- the regression coefficient for group identity was 0.770 (*t* = 22.946, *p* = 0.000 < 0.01), implying that group identity would have a significant positive relationship with negative emotions' communication. Hypothesis 3 does not hold.
- the regression coefficient for group efficacy regression coefficient was 0.806 (*t* = 25.953, *p* = 0.000 < 0.01), implying that group efficacy would have a significant positive relationship with negative emotions' communication. Hypothesis 4 does not hold.
- the regression coefficient for event stimulation was 0.825 (*t* = 23.039, *p* = 0.000 < 0.01), implying that group efficacy would have a significant positive relationship with negative emotions' communication. Hypothesis 5 does not hold.
- the regression coefficient for event publicness was 0.799 (*t* = 22.622, *p* = 0.000 < 0.01), implying that event publicness would have a significant positive relationship with negative emotions' communication. Hypothesis 6 does not hold.
- the regression coefficient for emotional contagion was 0.815 (*t* = 24.414, *p* = 0.000 < 0.01), implying that emotional contagion would have a significant positive relationship with negative emotions' communication. Hypothesis 7 does not hold.

**Figure 4:**
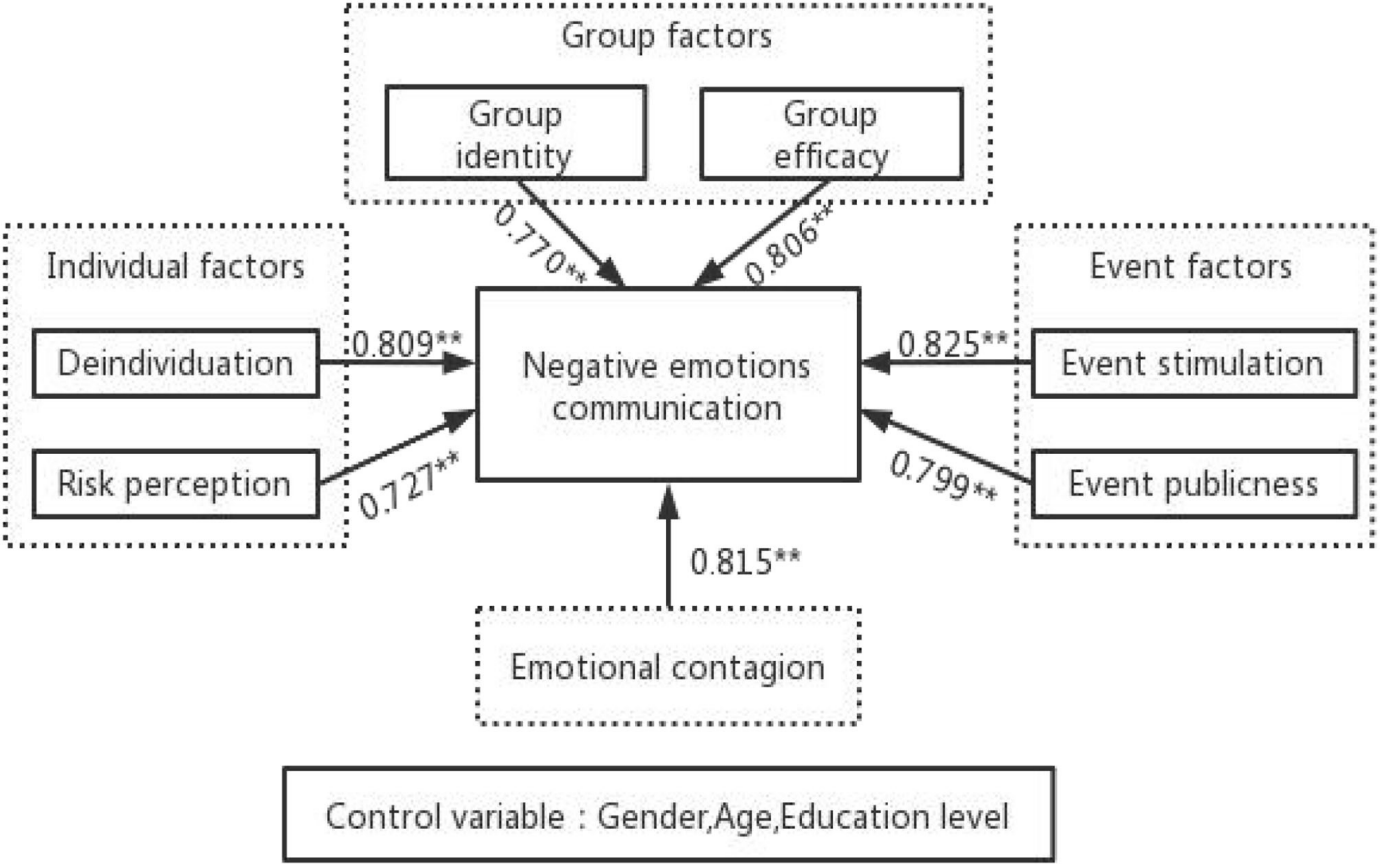
Model of the influencing factors on negative emotions communication. **p < 0.01.

With significant regression coefficients for all seven dimensions, all of which have a significant positive effect on the spread of negative emotions. Combined with the above analysis, all the hypotheses were not supported, except for hypothesis 8 and hypothesis 10. The graph of the model results is shown in Figure 4.

## Conclusion and Discussion

### Differences in Negative Emotions' Communication Among Users of Different Ages

From the statistical data, the group with stronger negative emotional communication is concentrated in 18–25 years old, followed by 26–30 years old, and the communication of negative emotion is weaker for users over 41 years old. There are two possible reasons for this concerning the social environment and online presentation.

The social roles played in the social environment, and the social status of different age groups in society differ in their expectations and stress levels. In mainstream sociological perspectives, social roles and self-presentation provide a way to understand individual social participation with its normative and pervasive nature, as well as interests and vocational needs. This involves a focus on subjects' experiences and patterns of response to complex and potentially conflicting normative structures (Smyth, 2021). The impact of school lockdown on life during the epidemic and the employment pressure brought about by corporate layoffs have put young people in a significant disconnect between expectations and reality. When they are overloaded with negative emotions, they are more willing to release their stress through external means.

From the perspective of Internet use and self-presentation, The age group in the 18–30 age bracket is the mainstay of the Internet platform and is more connected to social media. Online platforms are the easiest and fastest way to spread their emotions for those who use the Internet regularly (Keutler and McHugh, 2022). As a result, social media platforms show a stronger negative sentiment among the 18–30 age group than other age groups.

### Positive Effect Between Variables and Negative Emotions' Communication

The individual factor of deindividuation and risk perception has a significant positive effect on negative emotional communication. When the public's personal information is anonymized on social media platforms, individuals' topic engagement and positivity increase significantly (Vladimirou et al., 2021). Still, when the public is in a real social environment, they may behave more rationally out of the need to conform to the group and maintain their image. In terms of risk perception, individuals' negative emotions are influenced by external stimuli. When social environment makes them feel a strong sense of insecurity and uncertainty, it will intensify their negative emotional stimuli and thus trigger negative emotional communication (Savadori and Lauriola, 2022).

Group identity and group efficacy among the group factors have a significant positive effect on negative emotional communication. In this study, group identity refers to the tendency to feel the same emotions as others or agree with them. Group efficacy relates to users' perception that negative emotional expressions about hot events can solve existing problems during the epidemic (Salice and Miyazono, 2020). Based on observations of hot topics on Chinese social media platforms, this study found that users' emotional feedback mainly consisted of empathy for the vulnerable groups living under the epidemic and criticism of inappropriate government initiatives. At the same time, users felt that many reposts and comments on hotspots could urge the government to develop better emergency response strategies. This phenomenon suggests that individuals are aware that a group effort can transform some negative emotions into a booster for solving social problems.

Event stimulation and event publicness among the event factors had a significant positive impact on negative emotional communication. In this study, event stimulation refers to the conflict between the hot events in the COVID-19 epidemic and the universal values, such as the issue of culling pets during quarantine and the problem of treating patients with sudden illnesses. Internet users expressed their anger, condemnation, and grief about the events. Publicity of events is more inclined to the breadth of the ripple effect of events, such as the surge in the number of infected people and the long-term closed management. Therefore, negative emotional communication can be predicted by the nature of the hot events.

Emotional contagion has a significant positive effect on negative emotional communication. When social media platforms are flooded with negative expressions related to the epidemic, it reinforces users' perceptions and psychology, creating an emotional convergence effect. During the COVID-19 epidemic, fear of viral infection puts individuals in a sensitive state. Social events related to the epidemic were frequent, individual emotions gathered into group emotions due to the emotional contagion effect, and the Internet was collectively emotionally charged for a long time. As a result, when individuals are more susceptible to negative emotions, the communication of the negative emotion is also more intense. Improving users' ability to regulate emotions and awareness of the emotional contagion effect is a crucial step in the reconstruction and recovery of public mental health in the post-epidemic era.

In summary, during the particular epidemic period, all seven dimensions introduced in this study stimulate users' experience of negative emotions and inspire them to spread negative emotions on social media platforms. The regression coefficients for de-individuation, group efficacy, event stimulation, and emotional contagion were above 0.8, with more significant positive effects on negative emotional communication. It is worth noting that the statistical results from the two influencing factors of group efficacy and event publicness can help the public to break the stereotype of negative emotions. Psychologically, negative emotions are a phase of psychological states, the psychological motivations that induce negative emotional communication behavior include altruistic tendencies, and negative emotional communication behavior can reflect individual participation in public events. Negative emotional communication does not bring about a single outcome. Therefore, in sociology, negative emotions are classified as a social resource, which can be used appropriately to promote the solution of social problems. For example, in social public events, users make emotionally charged statements and form public opinion in cyberspace, triggering the attention of the relevant authorities and media, thus influencing the evolution of the event, which is a common situation where emotions act as a driving force in solving social problems. Based on this view, individuals should reasonably regulate negative emotions, and the social environment should not simply suppress negative emotions and terrorize them.

The research further confirms the existence of emotional contagion in social media. Emotional contagion deserves as much attention from the community as viral infection and information epidemics and is a potential disease under the epidemic. When individuals are aware of the emotional contagion effect, they can improve their ability to screen misinformation, thus mitigating the impact of negative emotions on themselves. Subsequent research on emotion improvement strategies can be targeted according to the influencing factors.

### Implications for Mitigating Negative Emotions Communication

A positive responsive attitude and a well-developed emergency response strategy from government departments are tranquilizers for the public sentiment (Huang et al., 2021). During the epidemic, the government should be quick to respond to public demands and social pain points to alleviate the public's psychological uncertainty in the face of unknown risks. Delay in responding can lead to false rumors being spread widely, and the public is frightened. In terms of response, a generalized or boilerplate approach will lead to public suspicion of the government and the perception that it has not effectively researched and solved the problem. The public will be socially rebellious and alienated from government decisions in the long run. At this particular time of the new epidemic, a society with shallow trust levels will be in an information epidemic and emotional war with rumors spreading and groups attacking each other. Therefore, the government and relevant departments should develop a systematic emergency response mechanism for the epidemic, with better handling of the way and content of responses and a more scientific design of measures to solve the problem.

The media should focus on science communication. The scientific knowledge about the epidemic can mitigate the extreme emotional tendencies of the public. The press can interview relevant experts on the interpretation of epidemic prevention and future development trends, helping the public to develop a more comprehensive understanding of virus, and reducing excessive fear (Albrecht et al., 2022). For example, the COVID-19 news section of the “Guokr” website has received a lot of attention from users by popularizing the scientific knowledge of the epidemic, such as whether clothes can absorb the virus and how to save oneself from excessive anxiety. In terms of reporting strategies, it is essential to grasp the laws of public sentiment. In the early stages of damaging sentiment outbreaks, the media should publish authoritative information promptly so that the truth is delivered faster than the speed at which extreme sentiment spreads. During the period of spreading negative emotions and in the later stages, the media should investigate the core demands of the public and care about the public interests of society so that the vulnerable groups and injustices in the epidemic are given attention. If the media chooses to remain silent during the outbreak in the face of events related to public morality and the interests of citizens, it will further ignite public sentiment and lead to irrational clustering behavior.

The rapid flow of emotions on social media platforms requires high media literacy and information screening skills. When faced with a public health emergency, it is normal to feel anxiety, fear, anger, and other negative emotions. Individuals should accept negative emotions and reduce the adverse effects of emotional accumulation and infection. Users can divert their attention and broaden the channels for releasing stress, avoiding over-reliance on social media, and focusing on self-development in real life.

In public health emergencies, the government should promote the construction of psychological assistance services and establish professional psychological assistance teams, such as online psychological counseling, psychological hotlines, and public service lectures (Rizzi et al., 2022). The combination of technology and psychology is also a new approach. During the epidemic, the “Anxin” team went to Wuhan to carry out psychological assistance work, based on the emotion tracking system, which provided an effective means of detecting and monitoring emotions. The public should also take the initiative to actively seek help when they realize that their psychological state is sub-healthy, change their misconceptions about emotions and mental illness, learn ways to improve their wellbeing and mediate their stress reactions under the guidance of experts.

### Limitations and Future Research

Despite these strengths, some limitations need to be underlined. From the research design perspective, this research explores the factors influencing negative emotional communication during the COVID-19 epidemic. The related studies are mostly text mining and sentiment analysis; the quantitative research in this area is not yet abundant, and the theoretical models selected are few. In the future, researchers can introduce new variables into the framework of the negative emotions' communication mechanism to improve the complexity of the framework and the accuracy of the model. At the questionnaire audience level, the coverage of the gender, age, and education variables were limited, resulting in a more concentrated sample. The questionnaire distribution process could be expanded to increase universality in the future. In terms of research methods, future quantitative research can be supplemented with interviews in qualitative research and consultation with psychologists and experts to propose more practical measures for psychological recovery and reconstruction of the individuals.

## Data Availability Statement

The original contributions presented in the study are included in the article/supplementary material, further inquiries can be directed to the corresponding author.

## Ethics Statement

Ethical review and approval was not required for the study on human participants in accordance with the local legislation and institutional requirements. Written informed consent from the patients/ participants or patients/participants legal guardian/next of kin was not required to participate in this study in accordance with the national legislation and the institutional requirements.

## Author Contributions

Both authors listed have made a substantial, direct, and intellectual contribution to the work and approved it for publication.

## Funding

This article is supported by the research results of the 2018 Guangdong Ordinary University Characteristic Innovation Project: Research on the Construction Path of Guangdong's New-type Think Tank Public Opinion Communication Power in the New Media Era (2018WTSCX195).

## Conflict of Interest

The authors declare that the research was conducted in the absence of any commercial or financial relationships that could be construed as a potential conflict of interest.

## Publisher's Note

All claims expressed in this article are solely those of the authors and do not necessarily represent those of their affiliated organizations, or those of the publisher, the editors and the reviewers. Any product that may be evaluated in this article, or claim that may be made by its manufacturer, is not guaranteed or endorsed by the publisher.
